# How to Deal with Paper Rejection

**DOI:** 10.3390/tomography12030031

**Published:** 2026-03-02

**Authors:** Emilio Quaia

**Affiliations:** Department of Radiology, University of Padova, Via Giustiniani 2, 35128 Padova, Italy; emilio.quaia@unipd.it; Tel.: +39-049-8212375

This editorial provides insights into the common situation of paper rejection, which must be managed by the authors. Rejection is part of the academic scientific process and there is a general need to clarify the best attitude towards paper rejection.

Sooner or later every author experiences paper rejection. We can easily say that rejection is part of academic life and that from each rejected scientific paper, a top scientific paper can finally thrive. Unfortunately, authors always consider the rejection of their work as a defeat and a true disappointment and, in those cases in which the authors believe that their paper rejection is unfair, that disappointment may become anxiety and even anger. In reality, it is not easy to emotionally deal with this. Moving past these psychological aspects, here are some suggestions to manage paper rejection.

First, paper rejection is not a verdict on your talent and should not be considered a personal attack. Do not take it personally: rejection is a standard part of academic life and does not define the worth of your research. Besides being the editor in chief of *Tomography*, I am also an author and some of my papers were and will be rejected.

Second, paper rejection is actually a judgment focalized to one single paper and not to the whole scientific production and career of an author. It is just a spark lasting a brief time and which will be forgotten pretty soon. All researchers, even the very best ones, have papers rejected. Even the very best papers get rejected. Part of the reason for this is that, in many cases, the peer review process is non-deterministic.

Third, an unfair paper rejection, while frustrating, is a hurdle often caused by poor communication, or inattentive reviewers. We know that all scientific journals are facing a true crisis in the scientific reviewing process [1] and reviewers are not always completely dedicated to their job and could make mistakes. If the decision was truly unwarranted, consider appealing with logical arguments and send a polite, evidence-based appeal to the editor. Always be respectful towards the editorial office, editor, and reviewers. Your courtesy and humility go a long way in winning a re-review. Point out the misunderstanding and provide appropriate clarification. Indicate that you have gathered new information and explain how it may reverse the original rejection.

Fourth, be honest with yourself and evaluate the rejection decision objectively, especially if the decision was a desk rejection mainly due to wrong journal selection or even lack of compliance with journal guidelines.

Fifth, we are fortunate to have plenty of scientific journals and if an appeal is unlikely to succeed, address the comments (even the unfair ones) to strengthen the manuscript and submit to a different journal. Extract any useful, constructive criticism from the reviews to improve the paper for future submissions [2].

Sixth, if your paper was rejected, do not try to modify your data to make them more attractive. Data fabrication, “making up data or results”, and data falsification, “manipulating research materials or omitting data or results”, are, besides plagiarism, true scientific sins and misconduct, which may jeopardize your entire academic career. I am aware that this suggestion may look obvious but many researchers, especially junior ones, fall into this mistake and may destroy their future career, becoming unreliable in the eyes of their colleagues. Anyone who engages in these behaviors is putting his or her scientific career at risk and such misconduct is treated very hard by the scientific community and by institutions.

Seventh, ask one of your colleagues, with specific expertise and not included in the author pool, to provide you with a frank assessment of your rejected paper. All authors believe that their paper is wonderful but sometimes we are not the best referee of ourselves.

Eighth, remember that there is a place for every paper and do not lose your hope of finding a suitable venue for your research.

Finally, we should consider rejection not as a defeat but as an opportunity to improve our manuscript and to improve our skill as authors.
